# Mechanical properties of an experimental resin based composite containing silver nanoparticles and bioactive glass

**DOI:** 10.12669/pjms.36.4.1913

**Published:** 2020

**Authors:** Amjad Hanif, Fazal Ghani

**Affiliations:** 1 Amjad Hanif, BDS, MSc. (UK). Assistant Professor, Department of Dental Materials, Peshawar Dental College, Peshawar, Pakistan; 2 Fazal Ghani, PhD (London), FDSRCPSGlasg, FRSM (UK), MSc (London), BDS. BSc (Pesh). Advanced PG Certificate in Clinical Education (RCPSGlasg), Head of Department of Prosthodontics, Dean, Post Graduate Dental Sciences Peshawar Dental College, Peshawar, Pakistan

**Keywords:** Resin based composites, Silver nanoparticle, Bioactive glass, Mechanical properties, Flexural Strength, Elastic modulus, Hardness

## Abstract

**Objective:**

To compare the elastic modulus, flexural strength, and hardness of an experimental resin based composite (RBC) with and without containing silver nanoparticles (AgNPs) and bioactive glass (BAG) with a commercially available RBC.

**Methods:**

This study was conducted, during the period August 2016-May 2018, at the Department of Dental Materials, Peshawar Dental College, Peshawar (Pakistan) and Department of Chemistry, University of Montreal, Canada. Test specimens made in the commercial RBC acted as Group-1 (G1). An experimental RBC containing 70 wt % filler content was synthesized. It was first used as such to prepare test specimens to act as the experimental control group (G2). This RBC was then modified by adding various amounts of BAG (5%, 10% and 15%) and a fixed amount of 0.009% AgNPs to use the so modified RBCs for preparing the test specimens to belong to three groups (G3, G4 & G5). The AgNPs had been synthesized in situ by reduction of salt during photo-polymerization. Flexural strength (FS), elastic modulus (EM) and Vickers hardness were determined using universal testing machine and hardness tester respectively. Data were analyzed using one-way ANOVA and Tukey post-hoc test.

**Results:**

Except for G3 restorations showing significantly lower mean FS value, the FS for those in the other groups were not significantly different (p>0.05). Elastic modulus of the experimental RBC restorations was though higher than those of the others but the difference was statistically insignificant (p>0.05). Reduced Vickers hardness values were documented for the restorations in the G4 and G5 compared to those in the G3 but again the difference was insignificant (p>0.05). Flexural strength and hardness values of the test specimens in the experimental RBCs were significantly lower than those made in the commercial hybrid RBC (p<0.05).

**Conclusion:**

BAG and AgNPs addition to the experimental RBC in the mentioned concentration adversely affected the tested mechanical properties.

## INTRODUCTION

Resin based composites (RBC) are frequently employed in modern dental practice to restore carious lesions.^1^ This is due to their better properties both in appearance and function.^2^ Several studies investigating RBCs have resulted in significant developments in filler and polymer matrix structures as well as manipulation and curing characteristics.^3^ The biofilm attached to the restorative materials or at the tooth-restoration border can cause recurrent caries, reducing the longevity and stability of the restoration.^3^ RBCs exhibit higher level of harboring of microorganism as compared to other restorative materials.^4^ The lack of antibacterial property of polymerized resin composites leads to increased bacterial attachment and plaque buildup on its surface than the other dental restorative materials including silver amalgam. The latter exhibit some antibacterial activity due to release of metal ions.^5^

Various antibacterial agents have been added to RBC to render them antibacterial.^6^ These include, quaternary ammonium dimethacrylate, quaternary ammonium polyethyleneimine nanoparticles, chlorhexidine and triclosan etc.^7^ Silver exhibits broad spectrum activity against fungi, bacteria and certain viruses. In order to reduce surface microbial biofilm, silver nanoparticles (AgNPs) are being applied as antimicrobials in biomaterials. The small sizes of AgNPs, facilitate a larger surface area to be coming in direct contact with microorganism and hence may be better interacting than large size particles.^3^ Furthermore, only a small amount of AgNPs if present in RBC is sufficient to make it strongly antibacterial, without significant alteration in the optical or mechanical properties of the RBC.^3^

Furthermore, since its discovery in the 1970s by Larry Hench, bioactive glasses (BAG) have been the focus of investigations as biomaterials for bone tissue substitution. BAG forms strong bond with both hard and soft tissues.^7^ Due to its unique re-mineralizing and antibacterial properties, fine particle BAG is generally introduced into various dentifrices to provide calcium and phosphorus to the tooth surface.^8^ Therefore, BAG may be a strategic repair element for tooth tissue if added to RBC restorative materials.^9^

Keeping with these findings, it was considered that to inculcate bioactivity and antimicrobial properties, it would be advantageous to add AgNPs and BAG to the RBC. However, a concern could be that these impregnations in the resin matrix, if not adequately bonded, could adversely affect the desired mechanical behavior of the RBC. Previously, the silver ions have been incorporated into the bioactive glass structure. However, we aim for a study whereby using silver separately from the BAG in the form of nanoparticles. Also knowing that some types of bioactive glasses have been tested before in RBC but in none, BAG 45S5 has been tested as we aim to do so for the first time in combination with silver nanoparticles, Therefore, the aim of this study was to see the effect, on mechanical properties, of the addition of both the AgNPs and BAG in the experimental RBCs.

## METHODS

The research protocol for the study was approved by the Board of Advanced Study & Research of the University (Riphah/26/17/011 March 03, 2016) as well as its publication by the Institutional Review Board (No. PRIME/IRB/2019-179). The experimental work was conducted during the period August 2016-May 2018), at the Department of Dental Materials, Peshawar Dental College, Peshawar (Pakistan) and Department of Chemistry, University of Montreal, Canada. The materials used for the synthesis and RBC modifications are given in Table-I. The experimental RBC was synthesized by mixing 1:1 mass ratio of bisphenol A-glycidyl methacrylate (Bis-GMA) and Tri-ethylene-glycol-dimethacrylate (TEGDMA) monomers with 0.4 wt% of camphoroquinone, 0.8 wt% of 4- dimethylaminobenzoic acid ethyl ether. In order to prepare 0.03% mass fraction of silver salt in resin, 10% solution of silver 2 ethyl hexanoate was prepared in 2-tert butyl amino ethyl methacrylate (TBAEMA). Then 1% of this solution was added to Bis GMA, TEGDMA resin. Based on a previous study9 fixed proportion of 0.009% of AgNPs were used in our experimental RBCs. Silica particles used in the study were synthesized using Stober method.^10^ with details given in Table II. Table-III presents of the relevant information of the various RBCs used in this study.

**Table-I:** Materials used in this study.

Materials	Manufacturer	Batch No
BisphenolGlycidyl methacrylate (Bis-GMA)	Sigma Aldrich USA	806-321
Triethylene glycol dimethacrylate (TEGDMA)	Sigma Aldrich USA	STBG35210V
Camphoroquinone (CQ)	Sigma Aldrich USA	A0097555
Ethyl-4-(dimethylamino) benzoate (EDMAB)	Alfa Aesar USA	MKCB6154
Silver 2 ethyl hexanoate	Strem Chemicals USA	A1918077
Bioactive Glass (45S5)	Denfotex Research Ltd UK	1608108
2-tert butyl amino ethyl methacrylate (TBAEMA)	SigmaAldrich, USA	MKCB2542V
Tetra ethyl orthosilicate (TEOS)	Sigma Aldrich, USA	MKBP8202V
Ammonium Hydroxide (NH_4_OH_)_	Sigma Aldrich, USA	45304
Nano silica (50nm)	Aerosol OX	154052345

**Table-II:** Reaction conditions for preparation of silica.

Target Size	Ethanol (ml)	Water (ml)	Ammonium hydroxide (ml)	TEOS* (ml)	Supplementary TEOS(ml)	Rate of addition ml / hour (g)	Mean size (nm)
1µm	250	40	25	15.5	31	2ml/hour	0.9-1µm

TEOS =Tetraethyl orthosilicate - Si (OH)_4_

**Table-III:** RBC groups with details of their contents.

Group 1 (G1)	Commercial resin composite(3M Filtek Z250XT)
Group 2 (G2)	Experimental resin composite (70 wt % filler and 0%BAG, 0%AgNPs)
Group 3 (G3)	Experimental resin composite (70 wt % filler and 5%BAG,0.009%AgNPs)
Group 4 (G4)	Experimental resin composite (70 wt % filler and 10%BAG,0.009%AgNPs)
Group 5 (G5)	Experimental resin composite(70 wt % filler and 15%BAG, 0.009%AgNPs)

To enhance the interfacial union between resin matrix and inorganic particles, the particles were surface treated with ϒ-MPS using a previous procedure.^11^ The silane (0.50±0.01g), the silica (5.0±0.05g), n-propylamine (0.1±0.01g) and solvent (100ml cyclohexane) were mixed for 30 minutes at room temperature and then for additional 30 minutes at 60±5°C. Rotary evaporator at 60±°C was used for removal of the byproducts and solvent. The powder obtained was then heated for one hour at 90±5°C in rotary evaporator and then dried at 80°C in a dry heat oven for about 20 hours.

The control experimental RBC (G2) was prepared by mixing the resin mixture with 70 wt% filler comprising of silica (60% synthesized silica and 9% commercial nano-silica, size 50nm (Aerosol OX50) zirconia particles 1% (size 1-5 µm). First both the resin mixture and filler particles were mixed manually in a plastic container. Then the material was transferred to three roll mill (Exakt, TRM, Norderstedt, Germany) to obtain a homogenous material. The control experimental RBC specimens (G2, n=6) were then made in it. The experimental RBC was then modified by adding 0.009% AgNPs and with the substitution of the silica particles with 5, 10 and 15 wt% of BAG-45S5 respectively (Table-III) and to use it for the fabrication of specimens (n=6 in each case) to belong to G3, G4 and G5 groups. Specimens (n=6) in the control group (G1) were also made in a commercially available RBC. Details of the various RBCs used in this study are given in Table-III.

Flexural strength (FS) and elastic modulus (EM) for the RBC specimens were determined using three-point bending test according to the technique mentioned in ANSI/ADA Specification No. 27-2009 (ISO 4049). Silicon rubber mould was made for making RBC specimens to have length, width and height of 25±0.1mm, 2±0.1mm, and 2±0.1mm respectively. RBC mix (paste) was inserted into the moulds by using stainless steel instrument. The specimens were covered on both sides (top and bottom) with a cellulose acetate matrix strip. A clear rigid microscope glass slide having thickness of about 1 mm was placed on top and bottom of the matrix strip. Mould surface was divided into three equal segments and were cured by overlapping radiations for 20 seconds with portable light cure unit (Dmetec Co., Ltd. Korea). The intensity of curing light was 850 mW/cm^2^. The intensity of light was periodically checked with digital radiometer (Liang ya Dental Equipment Co, Gouang Dong China). Excess material was removed with 600 grit silicon carbide paper and finally assessed for accuracy and appropriateness. Specimen were stored in distilled water at 37°C for 24 hours. Three-point bending test was conducted in universal testing machine (Instron 5565 USA) with a cross-head speed of 0.75mm/min until fracture of the RBC specimen occurred. The maximum load applied on the RBC specimen at the time of fracture was noted and the flexural strength (FS) in MPa was determined using the following equation. The elastic modulus was calculated automatically by the built in software (Blue Hill) used by the universal testing machine.

**FS = 3×L×D/2×w×h^2^**

Where **L** is the load (Newtons), **D** is the gap between supports (mm), **w** is width of the specimen (mm) and **h** is the height or thickness (mm) of the RBC specimen.

Mould for preparing the specimens for testing hardness was made from silicon rubber. Test specimen were disc shaped having 8 mm diameter and 2mm height or thickness. RBC paste was inserted into the mould and covered with cellulose acetate strips and glass slides as described earlier. Specimen surfaces were cured by radiation for about 20 seconds with portable LED light cure unit (Dmetec Co., Ltd. Korea). Excess material was removed with 400 grit silicon carbide paper and finally verified for their accuracy and appropriateness. Hardness tester (Tukon 2100, USA) was used to determine hardness. The micro hardness was determined by applying a 500g load through pyramidal diamond micro-indentor with a dwelling time of 10s using 20X magnification. A total of five square shaped depressions, one in the center and four at periphery were made in each specimen. The average indentation size determined the Vickers hardness number (VHN) by using the formula.

**HV= 1.854×F (g)/D^2^ micrometer**

Where **F** is the force used (g), **D** is the measurement of the indentation (micro meter), and **HV** is the hardness value.

SEM (FE-SEM, JEOLJSM-7400F, Japan) was employed to assess particle size, morphology and filler distribution. The size of silica particles was determined by using dynamic light scattering (DLS) (Nano-ZS Analyzer, Malvern Instruments, UK). Silver nanoparticles were characterized by using uv-vis spectrophotometer (Cary uv-vis Agilent technologies, USA) in the wavelength range 300-850nm.

The statistical package for social sciences (SPSS) software version 19 for windows was used for data analysis. Descriptive statistics of the data including the mean values ± S. Dev for FS, ME and VH was computed. Statistical analyses of the data were done using analysis of variance (ANOVA) and Tukey’s post hoc method for estimating the significance of the differences of the mean values between the groups of specimens made in the various RBC materials (*P* < 0.05).

## RESULTS

The flexural strength (FS) values of the various RBC specimens in the various groups are shown in Table-IV. One-way ANOVA / Tukey HSD post-hoc test for the data between various groups are shown in Table V. FS of RBC specimens groups (G2, G3, G4, and G5) was significantly less than those of G1 or the commercial RBC *(p< 0.001)*. It appears that the impregnation of AgNp-BAG in the RBC reduced flexural strength (FS) of test specimens in the G3 as compared to those in the G2 while the FS of G4, G5 were higher in comparison to those in the G2 (Table-IV and V).

**Table-IV:** Mean and standard deviation values for flexural strength, elastic modulus & vickers hardness of RBC specimens. Numbers in parenthesis are standard deviation values.

RBC Groups	Flexural Strength (MPa)	Elastic Modulus (GPa)	Vickers Hardness (VHN)
G1	138.33 (43.83)	9.84(1.69)	76.93 (4.60)
G2	73.49 (19.52)	6.60 (0.65)	52.7 (5.96)
G3	61.15 (15.63)	7.05 (0.50)	64.9 (19.72)
G4	77.75 (2.95)	7.08 (0.46)	43.17 (5.44)
G5	74.09 (8.091)	7.14 (0.68)	43.24 (6.31)

**Table-V:** Statistical analysis of the data between groups.

Comparison Groups	Flexural Strength Data	Elastic Modulus Data	VHN Data
G1-G2			
G1-G3	S	S	S
G1-G4			
G1-G5			
G2-G3			
G2-G4			
G2-G5	NS	NS	NS
G3-G4			
G3-G5	NS	NS	S
G4-G5	NS	NS	NS

S = Significant difference NS = Not Significant difference.

Mean values for the elastic modulus (EM) are presented in Table-IV and their statistical analyses in Table-V. The variations between EM values for the G1 were significantly different than those for the other groups *(p<0.001)*. Mean EM value for G2 specimen was low when compared to G1. However, the mean EM values for G3, G4, G5 were higher when compared to G2 (Table-V and VI). The statistical significance of these are given in Table-V).

The Data in Table-IV also depicts the Vickers hardness values of the various specimen groups. The mean hardness value for G2 was lower than those in the G1 (Table-IV). The mean hardness value was highest for specimens in the G3 in comparison to the specimens made in the other experimental RBC groups. The statistical significance of the differences between the mean hardness values are given in Table-V.

Scanning electron microscopy (SEM) revealed round shaped silica particles with the size of the synthesized silica particles determined by SEM and Dynamic Light Scattering (DLS) as 0.9-1.0µm. Uv-vis spectroscopy showed the absorption peak centered at about 428nm wavelength indicating round shape of the particles. Single peak also indicated small size (less than 20nm) of the AgNPs. In addition, silver salt solution dissolved in resin mixture displayed color change from transparent to light brown when exposed to visible light from dental light cure unit indicating the formation of AgNPs. The size of the BAG particles determined by DLS was found to be 512nm.

## DISCUSSION

Not surprisingly, the commercially available RBC specimens (G1) showed the highest flexural strength values among the RBC groups studied. This could be attributed to the higher percentage of the silica as filler content.^12^ This finding for the G1 is in agreement with those of others who have found that RBCs with higher filler content showed increased flexural strength and that the amount of filler was the leading factor even with different size and shape of the filler.^13^ There was slight increase in flexural strength values of the specimens in the G4 and G5 while those in the G3 showed lower values for the flexural strength. However, the differences were statistically insignificant. Korkut et al.^14^ also reported insignificant differences when 5% and 10% BAG was added to the RBC. However, a decreased strength was noted when the percentage of BAG was raised up to 30% filler mass fraction. The results of this study are also consistent with another study which also reported insignificant differences when the BAG content was 15% in the RBC.^13^ In another study, BAG added to RBC in concentration of 3%, 6%, 9% and 12% respectively showed decrease in flexural strength values with increasing proportion of BAG.^15^ Possible explanation for this behavior might be that at low concentration the BAG and AgNPs are well distributed but at higher concentration these particles tend to aggregate and lead to defects which ultimately deteriorates its mechanical properties.^13^ In the study of Khvostenko et al, the 10% Ag-BAG was incorporated into RBC by hand mixing. Their results showed that Ag-BAG impregnated RBC had 30% lower flexural strength than the control group. The observed decreased strength might be due to the air voids incorporated during mixing the flowable composite with Ag-BG glass. Hence, the procedure of fabrication could also be the reason for weak composite material.^15^ Our results for the flexural strength values of specimens in the G2, G3, G4, G5 approach to those reported for some commercial hybrid RBCs.^16^ However, the values are slightly below the minimum flexural strength requirement (80MPa) for Type-1 restorative composites and significantly less than the flexural strength requirement (150MPa) for Type-2 restorative composite resin recommended by ISO standard 4049.

Similar to flexural strength, commercial resin composite Z250 (G1) showed significantly higher elastic modulus when compared to rest of the groups. Elastic modulus values for the specimens in the G2, G3, G4, G5 ranged from about 6.60-7.14 GPa which approach those described for some conventional resin composites filled with hybrid fillers (Flexural modulus 5-25 GPa). Factors such as filler shape, type, amount etc. account for difference in values obtained.^16^ Also the range of techniques for determining the elastic modulus, difference in sample size, cross head speed and storage medium are some likely reasons for differing results.^17^ In our study, elastic modulus slightly improved after the replacement of silica by BAG and AgNPs in the G3, G4 and G5 when compared to G2. However, post hoc Tukey’s test showed that difference between G2, G3 G4 and G5 are insignificant. This indicates that the replacement of silica by BAG and AgNPs had minimal effect on the elastic modulus of resin composite. Possible explanation might be that the size and amount of additives incorporated into RBC resin composites did not disrupt the resin network and maintained the mechanical properties of the control samples.^16^

There is no agreement among researchers for a standard Vickers hardness value. However, a hardness value exceeding 50 (VHN) is considered optimum for RBC.^18^ Similar to flexural strength and elastic modulus, hardness value of commercial RBC (G1) was higher than those of the other groups. This might be due to more filler content,^13^ smaller size of filler, broader size distribution of filler and higher degree of conversion observed in case of the commercial RBC (Z250 XT G1). The replacement of silica by BAG and AgNPs had minimal influence on the hardness of experimental RBC (G2). Statistically significant difference in hardness values was observed between the specimens belonging to the G3 and G4, G5. A possible explanation given for this behavior might be that at higher concentration, these particles tend to aggregate and lead to defects which ultimately deteriorate its mechanical properties.^14^ RBC groups (G2 and G3) meet the requirement (50VHN) for hardness suggested by some researchers, while the G4 and G5 showed slightly lower hardness values than that recommended.^18^

### Limitations of the study

The mass fraction of filler, type of filler and the monomers used in the commercial RBC were different than the experimental RBCs used in this study. Moreover, the effect of replacement of silica by AgNPs and BAG on mechanical properties was not determined individually. Therefore, it was not possible to predict precisely the impact of individual components on the mechanical properties of experimental RBC.

## CONCLUSION

The mechanical properties for the specimens in the various experimental RBC groups were significantly lower than those made in the commercial RBC. However, the experimental RBCs could, still be useful in restoring class III, V lesions where stresses due to masticatory forces would not be the major concern. Further indications would be deciduous teeth requiring restorations.

### Authors` Contribution

**AH:** Conceived the idea, designed and conducted the research and prepared the first draft of the manuscript.

**FG**: Refined, perfected the experimental protocol, supervised and interpreted the data, reviewed and edited the final submitted and well as the subsequent revised manuscripts,agreed to be the corresponding author and is responsible for integrity of research.
